# Discovering dispersion: How robust is automated model discovery for human myocardial tissue?

**DOI:** 10.1007/s10237-025-02005-x

**Published:** 2025-08-27

**Authors:** Denisa Martonová, Sigrid Leyendecker, Gerhard A. Holzapfel, Ellen Kuhl

**Affiliations:** 1 Institute of Applied Mechanics, Friedrich-Alexander-Universität Erlangen-Nürnberg, Erlangen, Germany; 2 Institute of Applied Dynamics, Friedrich-Alexander-Universität Erlangen-Nürnberg, Erlangen, Germany; 3 Institute of Biomechanics, Graz University of Technology, Graz, Austria; 4 Department of Structural Engineering, Norwegian University of Science and Technology, Trondheim, Norway; 5 Department of Mechanical Engineering, Stanford University, Stanford, CA USA

**Keywords:** Automated model discovery, Constitutive neural network, Human myocardium, Fiber dispersion

## Abstract

Computational modeling has become an integral tool for understanding the interaction between structural organization and functional behavior in a wide range of biological tissues, including the human myocardium. Traditional constitutive models, and recent models generated by automated model discovery, are often based on the simplifying assumption of perfectly aligned fiber families. However, experimental evidence suggests that many fiber-reinforced tissues exhibit local dispersion, which can significantly influence their mechanical behavior. Here, we integrate the generalized structure tensor approach into automated material model discovery to represent fibers that are distributed with rotational symmetry around three mean orthogonal directions—fiber, sheet, and normal—by using probabilistic descriptions of the orientation. Using biaxial extension and triaxial shear data from human myocardium, we systematically vary the degree of directional dispersion and stress measurement noise to explore the robustness of the discovered models. Our findings reveal that up to a moderate dispersion in the fiber direction and arbitrary dispersion in the sheet and normal directions improve the goodness of fit and enable recovery of a previously proposed four-term model in terms of the isotropic second invariant, two dispersed anisotropic invariants, and one coupling invariant. Our approach demonstrates strong robustness and consistently identifies similar model terms, even in the presence of up to 7% random noise in the stress data. In summary, our study suggests that automated model discovery based on the powerful generalized structure tensors is robust to noise and captures microstructural uncertainty and heterogeneity in a physiologically meaningful way.

## Introduction

Computational modeling provides important insights into the intricate relationship between myocardial structure and mechanical function (Göktepe and Kuhl 2010; Baillargeon et al. 2014; Peirlinck et al. 2021; Martonová et al. 2022). In particular, various passive material models have been proposed and investigated to accurately characterize the mechanical properties of myocardial tissue. These models aim to capture the complex anisotropic organization of the myocardium, which is critical for effective cardiac function. In the myocardium, muscle fibers are arranged in a helical pattern, creating a three-dimensional architecture that supports the contraction and relaxation cycles of the heart. This arrangement allows for efficient twisting and squeezing motions necessary for effective blood pumping (Streeter et al. 1969; Holzapfel and Ogden 2009; Katz 2010; Holz et al. 2023). Myocardial fibers are arranged in layers of myocytes grouped together, so called sheets which exhibit a dynamic sliding behavior and contribute to ventricular deformation. Finally, to fully characterize the local orthotropic structure, we define the normal direction perpendicular to both fiber and sheet directions. Understanding this complex organization and possible uncertainties and anomalies in the microstructural architecture provide important insights into both normal physiology and pathological conditions.

Traditional constitutive models, and recently discovered models based on the constitutive neural networks (Linka et al. 2023; Martonová et al. 2024; Peirlinck et al. 2024), often assume a perfect local alignment of a family of fibers at a given location, simplifying the fully anisotropic nature of the biological tissue, in particular the myocardium.

This structural complexity necessitates the use of more advanced modeling techniques. Recent studies have explored how planar variations in fiber angles influence transversely isotropic model discovery for arteries (Vervenne et al. 2025), and studied similar effects in orthotropic textile structures (McCulloch and Kuhl 2024). Although these studies directly vary a single angle between two fiber families, various model frameworks exist to account for probabilistic three-dimensional fiber dispersion around a given mean fiber direction. One model framework assigns each individual fiber within a dispersion its own strain energy, and the fibers are dispersed around a mean preferred direction according to an angular density distribution (Lanir 1983). This model framework was later modified (Sacks 2003; Driesse et al. 2005; Holzapfel and Ogden 2015; Martonová et al. 2021) and is referred to as angular integration (AI). The approach is based on full integration over a unit sphere, so that all possible fiber directions according to a given probability density function are considered. However, computational efficiency concerns have led to the adoption of alternative approximations such as the generalized structure tensor approach (Gasser et al. 2006; Holzapfel et al. 2015; Niestrawska et al. 2016). The advantages of this model framework include: (i) It is an algebraic formulation and therefore easier to implement than the AI formulation, (ii) it allows for explicit analytical results for a range of different deformations, (iii) the numerical analysis is less demanding, and (iv) it is more accurate because the numerical integrations required for the AI approach always lead to computational errors, whereas such integrations are not required for the generalized structure tensor model. Studies have shown that the predictive powers of the two models, generalized structural tensor based and artificial intelligence based, are almost identical for a significant range of large deformations (Holzapfel and Ogden 2017). More recent modeling efforts for fiber dispersion in soft biological tissues focus on capturing the complex anisotropic behavior of heterogeneous fiber orientations and stretch distributions. An alternative affine distribution framework (Britt and Ehret 2023) improves numerical tractability while accounting for fiber stretch variability and anisotropy. A novel switchless constitutive model (Arvind and Kannan 2025) removes mechanical response discontinuities (Latorre 2016), thus enabling smoother and more stable simulations, as exemplified in arterial tissue models.

Due to its flexibility and efficiency to model heteregenous fiber dispersion, in the present study, we utilize the generalized structural tensor approach and incorporate fiber, sheet, and normal dispersions into our material model discovery. In particular, instead of assuming a perfect fiber, sheet, and normal alignment along one particular direction, we assume that these characteristic directions are locally distributed with some probability. Although the effects of myofiber dispersion on myocardial mechanics have been explored (Eriksson et al. 2013; Melnik et al. 2018; Guan et al. 2022), its impact on the discovery of material models remains insufficiently understood. Here, we use dispersed invariants to explore the influence of uncertainty in fiber architecture on model discovery using constitutive neural networks, which offer a physics-informed approach to modeling complex material behavior directly from data. By embedding principles such as objectivity, material symmetry, and thermodynamic consistency into the network architecture, constitutive neural networks achieve accurate and generalizable predictions (Linka et al. 2021; Linka and Kuhl 2023). Recent applications to both hyperelastic and inelastic materials, including human myocardium (Martonová et al. 2024), arterial tissue (Peirlinck et al. 2024; Vervenne et al. 2025), brain (Linka et al. 2023; St. Pierre et al. 2023), skin (Linka et al. 2023), or skeletal muscle (Holthusen et al. 2023), have demonstrated their effectiveness in capturing both elastic and time-dependent behavior of biological tissues. These models enable automated model discovery and robust parameter identification from experimental data.

In the present study, to evaluate the impact of aleatoric noise, we introduce variability to the measured stress data. A key question we address is whether fiber dispersion and aleatoric noise result in the discovery of fundamentally different models or solely in modified parameter values.

**Fig. 1:**
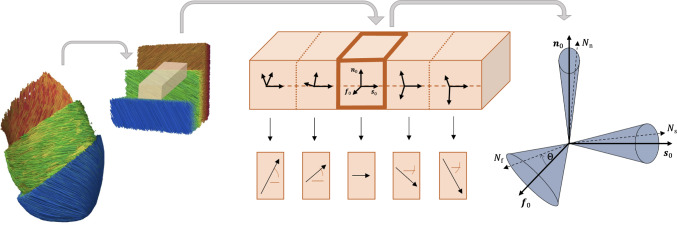
Fiber, sheet, and normal architecture in the left ventricle. Left and middle: Schematic representation of fiber architecture in left ventricle, rotating throughout the ventricular wall. Right: Schematic representation of the dispersion local in fiber, sheet, and normal directions. Fibers, sheets, and normals are assumed to be located inside the blue cone

We illustrate these effects through a case study on heart model discovery and compare the results with previously discovered models and parameters (Martonová et al. 2024) for given constant fiber, sheet, and normal orientations. To achieve this objective, we modify the architecture of the constitutive neural network to account for dispersions in the fiber, sheet, and normal directions and investigate the sensitivity of model discovery to both, the amount of the dispersion and the amount of noise in the stress–strain measurements. We train our network simultaneously on the biaxial extension and triaxial shear data (Sommer et al. 2015).

## Methods

### Continuum model

In continuum mechanics, a deformation map $\vec {\varphi }$ describes how material points move from their original (reference) configuration $\vec {X}$ to their current (deformed) configuration $\vec {x}=\vec {\varphi }(\vec {X})$ (Gurtin 1981; Holzapfel 2000). The deformation gradient ${\textbf{F}}$ of the map $\vec {\varphi }$ with respect to the undeformed coordinates $\vec {X}$ and its determinant *J* are defined as

1 $$\begin{aligned} {\textbf{F}}&= \nabla _{\varvec{X}} \vec {\varphi }, \qquad J= \det ({\textbf{F}}) > 0. \end{aligned}$$

Multiplying the deformation gradient with its transpose ${\textbf{F}}^\mathrm{{t}}$ from the left, we obtain the right Cauchy–Green deformation tensors ${\textbf{C}}= {\textbf{F}}^\mathrm{{t}} \cdot {\textbf{F}}$.

We demonstrate our approach using human myocardial tissue, modeled as perfectly incompressible orthotropic material with three distinct structural directions. We adopt the standard orthotropic architecture of the myocardium defined by myofiber, sheet, and sheet–normal directions (LeGrice et al. 1995; Usyk et al. 2000; Holzapfel and Ogden 2009), denoted by $\vec {f}_0$, $\vec {s}_0$ and $\vec {n}_0$, respectively. This architecture effectively represents the laminar structure observed in histology and diffusion tensor imaging, and we schematically illustrate it in Fig. 1. We introduce nine invariants to describe the deformation (Spencer 1984; Holzapfel and Ogden 2009), three standard isotropic invariants $I_1$, $I_2$, $I_3$, three anisotropic invariants characterizing the stretches, $I_\mathrm{{4f}}$, $I_\mathrm{{4s}}$, $I_\mathrm{{4n}}$, and three mixed coupling invariants, $I_\mathrm{{8fs}}$, $I_\mathrm{{8fn}}$, $I_\mathrm{{8sn}}$,

2 $$\begin{aligned} \begin{array}{llllll} I_1 & =& {\textbf{C}}: {\textbf{I}} & I_2& =& \frac{1}{2} \; [ I_1^2 - {\textbf{C}}:{\textbf{C}} ]\\ I_3& =& \det ({\textbf{C}}) = J^2 & I_\mathrm{{4f}}& =& {\textbf{C}}: [\vec {f}_0 \otimes \vec {f}_0]\\ I_\mathrm{{4s}} & =& {\textbf{C}}: [\vec {s}_0 \otimes \vec {s}_0] & I_\mathrm{{4n}} & =& {\textbf{C}}: [\vec {n}_0 \otimes \vec {n}_0]\\ I_\mathrm{{8fs}} & =& {\textbf{C}}:\textrm{sym} (\vec {f}_0 \otimes \vec {s}_0) & I_\mathrm{{8fn}} & =& {\textbf{C}}:\textrm{sym} (\vec {f}_0 \otimes \vec {n}_0)\\ I_\mathrm{{8sn}} & =& {\textbf{C}}:\textrm{sym} (\vec {s}_0 \otimes \vec {n}_0) . \end{array} \end{aligned}$$

where ${\textbf{I}}$ is the identity tensor. Under our assumption of perfect incompressibility, the third invariant equals one, $I_3=J^2=1$.

### Material model discovery with constitutive neural networks

**Fig. 2:**
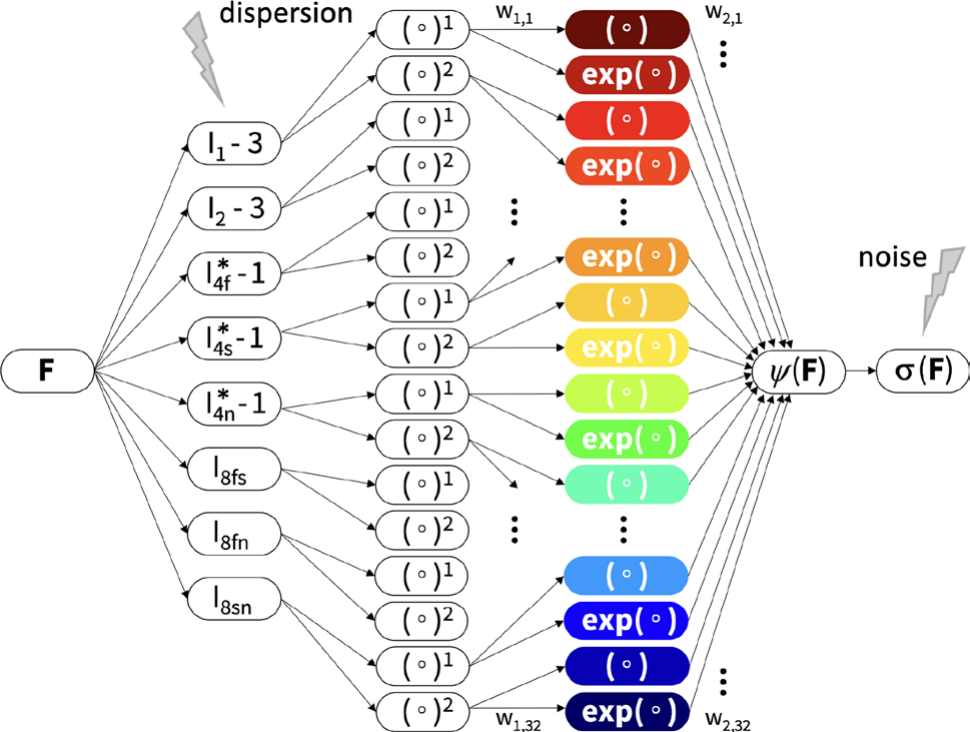
Orthotropic, perfectly incompressible constitutive neural network accounting for fiber, sheet, and normal dispersion. Eight invariants $I_1, I_2, I_\mathrm{{4f}}^*,I_\mathrm{{4s}}^*,I_\mathrm{{4n}}^*, I_\mathrm{{8fs}}, I_\mathrm{{8fn}}, I_\mathrm{{8sn}}$ serve as network input, while a scalar-valued free energy function $\psi$, depending on these eight invariants, is the network output. Cauchy stress components are derived from the discovered free energy function $\psi$. The displayed feed-forward partially connected network contains two hidden layers. In the first layer, the corrected eight invariants are raised to the first and second powers, $(\circ )$ and $(\circ )^2$, while the identity $(\circ )$ and exponential functions $(\mathrm{{exp}(\circ )}$ operate on these values

To autonomously discover the most suitable passive material model for human myocardial tissue with probabilistic fiber, sheet, and normal orientations, we use constitutive neural networks as our model discovery framework (Linka et al. 2021; Linka and Kuhl 2023). In particular, we select an orthotropic constitutive neural network made up of two hidden layers and 32 nodes with built-in incompressibility and polyconvexity of the free energy function $\psi$, being the network output (Martonová et al. 2024; Holzapfel and Ogden 2009). As shown in Fig. 2, eight invariants serve as network inputs. This network architecture is therefore capable of discovering up to $2^{32}$ possible models. In the present work, we include uncertainty in both network input and network output, to better reflect realistic experimental data.

#### Network input uncertainty—fiber dispersion

We follow the generalized structural tensor approach (Gasser et al. 2006) to include fiber, sheet, and normal dispersion into our network. This approach is based on a symmetric generalized structural tensor ${\textbf{H}}_i$ for each fiber family *i*, defined as

3 $$\begin{aligned} {\textbf{H}}_i = \frac{1}{4\pi } \int _{{\mathcal {S}}^2} \rho _i(\vec {N}_i(\varTheta ,\varPhi ))\vec {N}_i(\varTheta ,\varPhi ) \otimes \vec {N}_i(\varTheta ,\varPhi ) \, {\mathrm{{d}}} \omega , \end{aligned}$$

where ${\mathcal {S}}^2=\{\vec {N}_i:|\vec {N}_i|=1\}$ is the unit sphere, ${\mathrm{{d}}}\omega =\sin \Theta {\mathrm{{d}}} \varTheta \, {\mathrm{{d}}} \varPhi$, $\rho _i(\vec {N}_i(\varTheta ,\varPhi ))$ is a probability density function, and $\vec {N}_i$ is any unit vector in three-dimensional Eulerian space, i.e., $\varvec{N}_i$ denotes a possible fiber direction within fiber family *i*, and $\rho _i(\vec {N}_i(\varTheta ,\varPhi )) {\mathrm{{d}}} \omega$ represents the normalized number of fibers with orientations within the intervals $[\varTheta , \varTheta +{\mathrm{{d}}}\varTheta ], [\varPhi , \varPhi +{\mathrm{{d}}}\varPhi ]$ that fulfills the symmetry condition $\rho _i(\vec {N}_i)=\rho (\vec {-N}_i)$ (Gasser et al. 2006).

Now, we assume axisymmetric distributions for each family of undeformed fibers with the mean direction $\varvec{i}_0$. The density function is then independent of $\varPhi$ and simplifies to $\rho _i(\vec {N}_i(\varTheta ,\varPhi )) \approx \rho _i(\varTheta )$. For a given family of fibers, the fibers are distributed with rotational symmetry about a mean referential direction represented by a unit vector $\vec {i}_0$; the generalized structural tensor approach in Eq. (3) simplifies to

4 $$\begin{aligned}&{\textbf{H}}_i = \kappa _i{\textbf{I}} + [\,1-3\kappa _i] \vec {i}_0\otimes \vec {i}_0 \nonumber \\&\text {with} \quad \kappa _i=\frac{1}{4}\int _{0}^\pi \rho _i(\varTheta ) \sin ^3 \varTheta \, {\mathrm{{d}}} \varTheta , \end{aligned}$$

where $\kappa _i \in [0,1/3]$ is the dispersion parameter. In the following, we consider three fiber families, fibers, sheets, and normals, $i\in \{\textrm{f,s,n}\}$, and we assume that the orientations of these fiber families follow a modified $\pi$-periodic von Mises distribution. The probability density function centered at $\varTheta =0$ is then given by

5 $$\begin{aligned}&\rho _i(\varTheta ) = 4 \sqrt{\frac{b_i}{2\pi }} \frac{\exp \left\{ b_i\cos (2\varTheta ) \right\} }{\text {erfi} \left( \sqrt{2b_i} \right) }\nonumber \\&\text {such that} \quad \frac{1}{4\pi } \int _{\omega } \rho (\varTheta ) \, {\mathrm{{d}}} \omega = 1, \end{aligned}$$

with a concentration parameter $b_i>0$ and an imaginary error function $\text {erfi}(x) = -i \, \text {erf}(x)$, with

6 $$\begin{aligned} \text {erf}(x)=\frac{2}{\sqrt{\pi }}\int _0^x\exp (-t^2) {\mathrm{{d}}}t. \end{aligned}$$

**Fig. 3:**
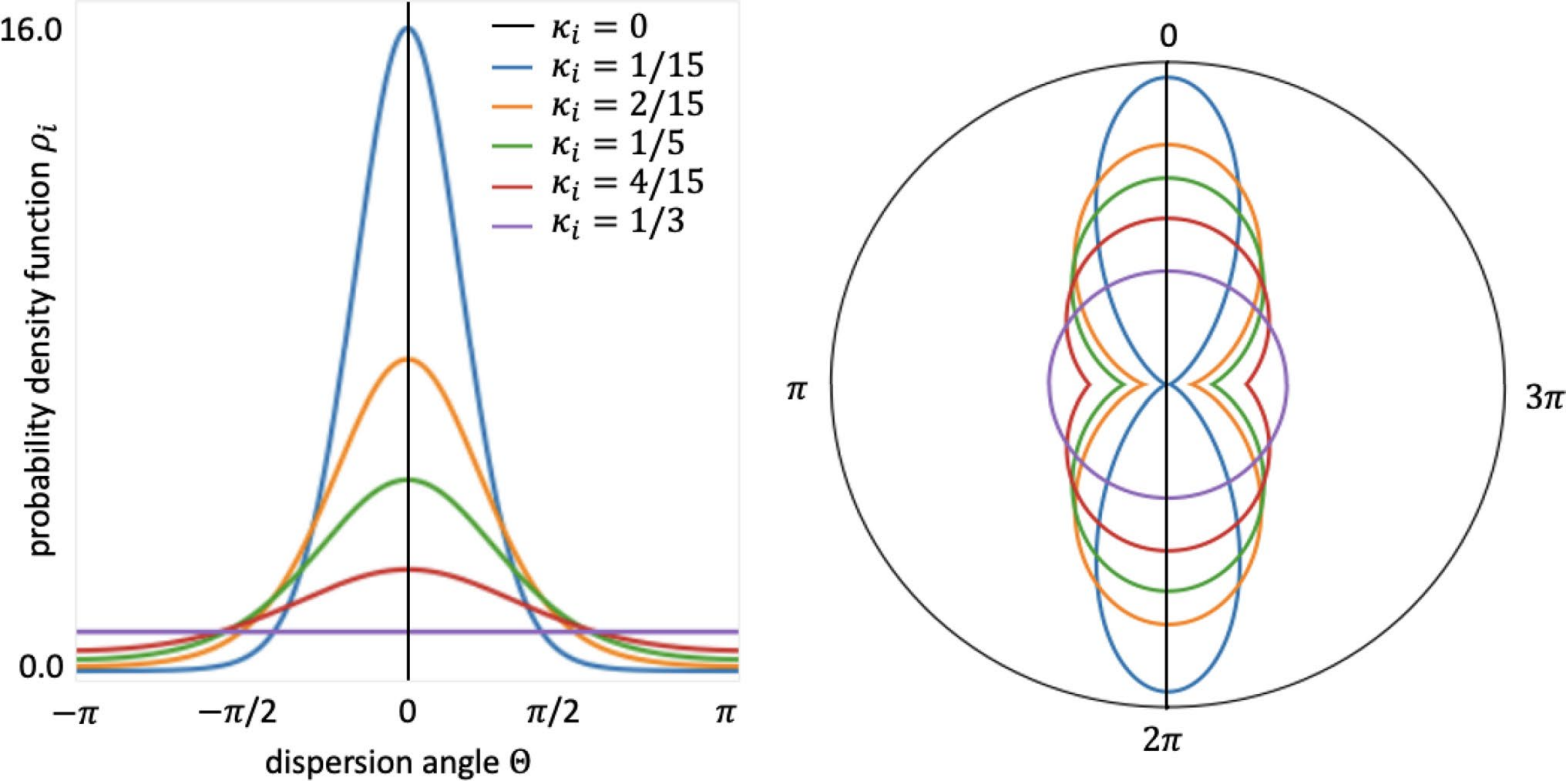
Modified von Mises probability density function for the dispersion angle $\varTheta$. Left: two-dimensional probability density function $\rho _i$ for different dispersion parameters $\kappa _i$. Right: planar schematic representation of possible fiber, sheet, and normal directions for a given dispersion parameter $\kappa _i$

We can interpret this distribution as a projection of the normal distribution onto the unit sphere (Fisher et al. 1987; Gasser et al. 2006). In particular, for $\kappa _i=0$, we recover a perfect fiber alignment along the mean direction $\vec {i}_0$, whereas for $\kappa _i=1/3$, the fibers are distributed isotropically within the sphere, as schematically shown in Fig. 3.

Using the generalized structural tensor approach, we modify the three anisotropic invariants $I_\mathrm{{4f}}$, $I_\mathrm{{4s}}$, $I_\mathrm{{4n}}$, and obtain the following dispersed invariants for our network input,

7 $$\begin{aligned} \begin{array}{lllllllll} I_\mathrm{{4f}}^* & =& {\textbf{C}}: {\textbf{H}}_\textrm{f} & =& \kappa _\textrm{f} I_1 & +& [\, 1-3\kappa _\textrm{f}]\, I_\mathrm{{4f}},\\ I_\mathrm{{4s}}^* & =& {\textbf{C}}: {\textbf{H}}_\textrm{s} & =& \kappa _\textrm{s} I_1 & +& [\, 1-3\kappa _\textrm{s}]\, I_\mathrm{{4s}},\\ I_\mathrm{{4n}}^* & =& {\textbf{C}}: {\textbf{H}}_\textrm{n} & =& \kappa _\textrm{n} I_1 & +& [\, 1-3\kappa _\textrm{n}] \, I_\mathrm{{4n}} . \end{array} \end{aligned}$$

Following previous studies (Eriksson et al. 2013; Guan et al. 2022), we solely account for the dispersion in the fourth invariants. We note, though, that it is also possible to account for dispersion in the modified coupling $I_8$-like invariant, see (Melnik et al. 2018). However, the softening effect of this coupled dispersed invariant was shown to be smaller than for the dispersed fourth invariants and its physical interpretation remains largely unexplored.

#### Network output uncertainty—added Gaussian noise

To investigate the influence of aleatoric uncertainty on the discovered model and its parameters, we systematically add varying levels of Gaussian noise, $\epsilon _\mathrm{{k}}$, $\mathrm{{k}} \in \{0.03,0.05,0.07,0.1\}$, to the measured values of the Cauchy stress components $\sigma _\mathrm{{ij}}$,

8 $$\begin{aligned} \sigma _\mathrm{{ij}} = \sigma _\mathrm{{ij}} + \epsilon _\mathrm{{k}} \quad \text {with} \quad \epsilon _\mathrm{{k}} \sim \mathrm{{k}} \, {\mathcal {N}}(0, \mathrm{{std}}_{\sigma _\mathrm{{ij}}}), \end{aligned}$$

where $\mathrm{{std}}_{\sigma _\mathrm{{ij}}}$ is the standard deviation of the measured Cauchy stresses for a given deformation mode. We refer to Table 1 in (Martonová et al. 2024) for the corresponding stress values.

#### Neural network architecture

Figure 2 showcases the architecture of our neural network with its two hidden layers and 32 nodes. The network assumes perfect incompressibility and takes eight input invariants: two isotropic invariants $I_1$ and $I_2$; three anisotropic invariants that account for fiber, sheet, and normal dispersion $I_\mathrm{{4f}}^*,I_\mathrm{{4s}}^*,I_\mathrm{{4n}}^*$; and three coupling invariants $I_\mathrm{{8fs}}, I_\mathrm{{8fn}}, I_\mathrm{{8sn}}$. The output is a scalar-valued free energy function $\psi$. In the first layer, the network generates the powers $(\circ )$ and $(\circ )^2$ of the corrected input invariants. In the second layer, the network applies the identity function $(\circ )$ and the exponential function $(\textrm{exp}(\circ ))$ to these values. This allows us to explicitly express the free energy function $\psi$,

9 $$\begin{aligned} \begin{array}{llllllllllll} \psi & =& w_{1,1} & w_{2,1} & [ I_1& - 3 ] & +& w_{2,2} & [ \, \exp ( w_{1,2} & [ I_1& -3 ]& ) - 1] \\ & +& w_{1,3} & w_{2,3} & [ I_1& - 3 ]^2 & +& w_{2,4} & [ \, \exp ( w_{1,4} & [ I_1& -3 ]^2& ) - 1] \\ & +& w_{1,5} & w_{2,5} & [ I_2& - 3 ] & +& w_{2,6} & [ \, \exp ( w_{1,6} & [ I_2& -3 ]& ) - 1] \\ & +& w_{1,7} & w_{2,7} & [ I_2& - 3 ]^2 & +& w_{2,8} & [ \, \exp ( w_{1,8} & [ I_2& -3 ]^2& ) - 1] \\ & +& w_{1,9} & w_{2,9} & [ {I}_\mathrm{{4f}}^*& - 1 ] & +& w_{2,10} & [ \, \exp ( w_{1,10} & [ {I}_\mathrm{{4f}}^*& -1 ]& ) - 1] \\ & +& w_{1,11} & w_{2,11} & [ {I}_\mathrm{{4f}}^*& - 1 ]^2 & +& w_{2,12} & [ \, \exp ( w_{1,12} & [ {I}_\mathrm{{4f}}^*& -1 ]^2& ) - 1] \\ & +& w_{1,13} & w_{2,13} & [ {I}_\mathrm{{4s}}^*& - 1 ] & +& w_{2,14} & [ \, \exp ( w_{1,14} & [ {I}_\mathrm{{4s}}^*& -1 ]& ) - 1] \\ & +& w_{1,15} & w_{2,15} & [ {I}_\mathrm{{4s}}^*& - 1 ]^2 & +& w_{2,16} & [ \, \exp ( w_{1,16} & [ {I}_\mathrm{{4s}}^*& -1 ]^2& ) - 1] \\ & +& w_{1,17} & w_{2,17} & [ {I}_\mathrm{{4n}}^*& - 1 ] & +& w_{2,18} & [ \, \exp ( w_{1,18} & [ {I}_\mathrm{{4n}}^*& -1 ]& ) - 1] \\ & +& w_{1,19} & w_{2,19} & [ {I}_\mathrm{{4n}}^*& - 1 ]^2 & +& w_{2,20} & [ \, \exp ( w_{1,20} & [ {I}_\mathrm{{4n}}^*& -1 ]^2& ) - 1] \\ & +& w_{1,21} & w_{2,21} & [ I_\mathrm{{8fs}}& \;] & +& w_{2,22} & [ \, \exp ( w_{1,22} & [ I_\mathrm{{8fs}}& \;]& ) - 1] \\ & +& w_{1,23} & w_{2,23} & [ I_\mathrm{{8fs}}& \;]^2 & +& w_{2,24} & [ \, \exp ( w_{1,24} & [ I_\mathrm{{8fs}}& \;]^2& ) - 1] \\ & +& w_{1,25} & w_{2,25} & [ I_\mathrm{{8fn}}& \;] & +& w_{2,26} & [ \, \exp ( w_{1,26} & [ I_\mathrm{{8fn}}& \;]& ) - 1] \\ & +& w_{1,27} & w_{2,27} & [ I_\mathrm{{8fn}}& \;]^2 & +& w_{2,28} & [ \, \exp ( w_{1,28} & [ I_\mathrm{{8fn}}& \;]^2& ) - 1] \\ & +& w_{1,29} & w_{2,29} & [ I_\mathrm{{8sn}}& \;] & +& w_{2,30} & [ \, \exp ( w_{1,30} & [ I_\mathrm{{8sn}}& \;]& ) - 1] \\ & +& w_{1,31} & w_{2,31} & [ I_\mathrm{{8sn}}& \;]^2 & +& w_{2,32} & [ \, \exp ( w_{1,32} & [ I_\mathrm{{8sn}}& \;]^2& ) - 1]. \end{array} \end{aligned}$$

Due to the incompressibility assumption, the free energy is modified by the hydrostatic pressure term *p*, yielding $\psi = \psi - p \, [J-1]$. The corrections for the invariants values by one and three ensure that $\psi ({\textbf{F}}={\textbf{I}})=0$ is satisfied. We note that we only activate the dispersed fourth invariants terms, ${I}_\mathrm{{4f}}^*$, ${I}_\mathrm{{4\,s}}^*$, ${I}_\mathrm{{4n}}^*$, if the fibers, sheet, and normal directions are under tension, i.e., if ${I}_\mathrm{{4f}}$, ${I}_\mathrm{{4\,s}}$, ${I}_\mathrm{{4n}} \ge ~1.$ This tension–compression switch is applied in all directions to prevent non-physiological compressive stiffening (Holzapfel and Ogden 2009; Göktepe and Kuhl 2010; Holzapfel and Ogden 2017). For the eighth coupling invariants, ${I}_\mathrm{{8fs}}$, ${I}_\mathrm{{8fn}}$, ${I}_\mathrm{{8sn}}$, in the undeformed configuration, the values are zero and can be used as such. Notably, these coupling invariants are sign-sensitive with respect to the fiber, sheet, and normal directions and are therefore not strictly invariant (Holzapfel and Ogden 2009; Melnik et al. 2018). However, when training the network with experiments specified in Sect. 2.3, the sign always remains positive. Following standard arguments of thermodynamics, we obtain the Cauchy stress from the free energy function $\psi$ in Eq. (9) as

10 $$\begin{aligned} \, \varvec{\sigma }&= \frac{\partial \psi }{\partial {\textbf{F}}} \cdot {\textbf{F}}^{\mathrm{{t}}} - p \, {\textbf{I}} =\sum _{k\in {\mathcal {I}}} \frac{\partial \psi }{\partial I_k}\frac{\partial I_k}{\partial {\textbf{F}}} \cdot {\textbf{F}}^\mathrm{{t}}- p \, {\textbf{I}} , \end{aligned}$$

where ${\mathcal {I}}=\{I_1, I_2, I_\mathrm{{4f}}^*,I_\mathrm{{4\,s}}^*,I_\mathrm{{4n}}^*,I_\mathrm{{8fs}},I_\mathrm{{8fn}}, I_\mathrm{{8sn}}\}$ is the set of network input invariants. Analytical expressions for the free energy derivatives with respect to the eight invariants depend on the network weights and are documented in prior work (Martonová et al. 2024). The derivatives of these invariants with respect to the deformation gradient follow as

11 $$\begin{aligned} \begin{array}{llllll} {\partial _{{\textbf{F}}} I_1} & = 2 & {\textbf{F}} \\ {\partial _{{\textbf{F}}} I_2} & = 2 & \left[ I_1 {\textbf{F}} - {\textbf{F}} \cdot {\textbf{F}}^T \cdot {\textbf{F}} \right] \\ {\partial _{{\textbf{F}}} I_\mathrm{{4f}}^*} & = 2 & {\textbf{F}} \cdot \left[ \kappa _\textrm{f}{\textbf{I}} + (1-3\kappa _\textrm{f})\vec {f}_0 \otimes \vec {f}_0\right] \\ {\partial _{{\textbf{F}}} I_\mathrm{{4s}}^*} & = 2 & {\textbf{F}} \cdot \left[ \kappa _\textrm{s}{\textbf{I}} + (1-3\kappa _\textrm{s})\vec {s}_0 \otimes \vec {s}_0\right] \\ {\partial _{{\textbf{F}}} I_\mathrm{{4n}}^*} & = 2 & {\textbf{F}} \cdot \left[ \kappa _\textrm{n}{\textbf{I}} + (1-3\kappa _\textrm{n})\vec {n}_0 \otimes \vec {n}_0\right] \\ {\partial _{{\textbf{F}}} I_\mathrm{{8fs}}} & = & {\textbf{F}} \cdot \left[ \vec {f}_0 \otimes \vec {s}_0 + \vec {s}_0 \otimes \vec {f}_0 \right] \\ {\partial _{{\textbf{F}}} I_\mathrm{{8fn}}} & = & {\textbf{F}} \cdot \left[ \vec {f}_0 \otimes \vec {n}_0 + \vec {n}_0 \otimes \vec {f}_0 \right] \\ {\partial _{{\textbf{F}}} I_\mathrm{{8sn}}} & = & {\textbf{F}} \cdot \left[ \vec {s}_0 \otimes \vec {n}_0 + \vec {n}_0 \otimes \vec {s}_0 \right] . \end{array} \end{aligned}$$

### Mechanical experiments used for training

Finally, we evaluate the stresses for the specific experimental loading modes. Motivated by our previous findings (Martonová et al. 2024), we train our dispersed-invariant network from Fig. 2 simultaneously with triaxial shear and biaxial extension data from human myocardial tissue (Sommer et al. 2015). In the following, the subscripts, $\mathrm{{f}},$ $\mathrm{{s}},$ $\mathrm{{n}}$, are associated with the fiber, sheet, and normal directions, respectively, and are used to denote the corresponding stretches $\lambda _\textrm{i}$ and shear strains $\gamma _\mathrm{{ij}},$ $\textrm{i,j}\in \{f,s,n\}.$ For all six triaxial shear experiments, the three stretches remain constant and equal to one, i.e., $\lambda _\mathrm{{f}} = \lambda _\mathrm{{s}} = \lambda _\mathrm{{n}} \equiv 1$. During triaxial shear testing in the ij-plane along the j-direction, only the shear strain $\gamma _\mathrm{{ij}}$ becomes nonzero, while all other shear strains remain zero. As a result, each of the six tests yields two nonzero shear stress components, $\sigma _\mathrm{{ij}} = \sigma _\mathrm{{ji}} \ne 0,$ in particular

12 $$\begin{aligned} \begin{array}{lll} \displaystyle {\sigma _\mathrm{{ij}} = 2 \gamma _\mathrm{{ij}} \left[ \frac{\partial \psi }{\partial I_1} \right. } & \displaystyle {\left. + \frac{\partial \psi }{\partial I_2} + (1-2\kappa _i) \frac{\partial \psi }{\partial I_\mathrm{{4i}}^*} \right. \;\;}\\ & \displaystyle {\left. + \kappa _j \frac{\partial \psi }{\partial I_\mathrm{{4j}}^*} + \frac{1}{2} \frac{\partial \psi }{\partial I_\mathrm{{8ij}}} \right] } & \displaystyle {=\sigma _\mathrm{{ji}}}. \end{array} \end{aligned}$$

We note that due to the fiber, sheet, and normal dispersions, $\kappa _i>0,$ both fourth invariants, $I_\mathrm{{4i}}^*$ and $I_\mathrm{{4j}}^*,$ contribute to the shear stress $\gamma _\mathrm{{ij}},$ whereas in the non-dispersed case, $\kappa _i=\kappa _j=0,$ only the invariant $I_\mathrm{{4i}}^*$ appears in Eq. (12).

For the biaxial extension tests, we consider five different ratios of fiber and normal stretches $(\lambda _\mathrm{{f}} \ge 1) : (\lambda _\mathrm{{n}} \ge 1)$, namely 1:1, 1:0.5, 1:0.75, 0.5:1, 0.75:1. The remaining sheet stretch is computed from the incompressibility condition as $\lambda _\mathrm{{s}} = 1/[\lambda _\mathrm{{f}}\lambda _\mathrm{{n}}]\le 1$ and all shear strains vanish. We further assume a zero stress condition throughout the sample thickness, such that the condition $\sigma _\mathrm{{ss}}=0$ holds. For the hydrostatic pressure *p* in (10), we obtain

13 $$\begin{aligned} p=2\frac{1}{\lambda ^2_s}\frac{\partial \psi }{\partial I_1} + 2\left[ \frac{1}{\lambda ^2_f}+\frac{1}{\lambda ^2_n}\right] \frac{\partial \psi }{\partial I_2} \,. \end{aligned}$$

The nonzero normal stresses take the following form:

14 $$\begin{aligned} \begin{array}{llllllllllll} \displaystyle {\sigma _\mathrm{{ff}}} \displaystyle & {=}& \displaystyle {2\frac{\partial \psi }{\partial I_1}} \displaystyle {[\lambda _\mathrm{{f}}^2-\lambda _s^2]} \displaystyle {+} \displaystyle {2\frac{\partial \psi }{\partial I_2}} \displaystyle {[\lambda ^2_\mathrm{{f}} - \lambda _\mathrm{{s}}^2] \, \lambda _\mathrm{{n}}^2}\\ & \displaystyle {+}& 2\lambda ^2_\mathrm{{f}}\left[ \displaystyle {\frac{\partial \psi }{\partial I_\mathrm{{4f}}^*}} (1-2\kappa _\textrm{f}) + \displaystyle {\frac{\partial \psi }{\partial I_\mathrm{{4n}}^*}} \kappa _\textrm{n}\right] ,\\ \displaystyle {\sigma _\mathrm{{nn}}} & \displaystyle {=} & \displaystyle {2\frac{\partial \psi }{\partial I_1}} \displaystyle {[\lambda _\mathrm{{n}}^2-\lambda _s^2]} \displaystyle {+} \displaystyle {2\frac{\partial \psi }{\partial I_2}} \displaystyle {[\lambda ^2_\mathrm{{n}} - \lambda _\mathrm{{s}}^2] \, \lambda _\mathrm{{n}}^2}\\ & \displaystyle {+}& 2\lambda ^2_\mathrm{{n}}\left[ \displaystyle {\frac{\partial \psi }{\partial I_\mathrm{{4n}}^*}} (1-2\kappa _\textrm{n}) + \displaystyle {\frac{\partial \psi }{\partial I_\mathrm{{4f}}^*}} \kappa _\textrm{f}\right] . \end{array} \end{aligned}$$

In Eqs. (12) and (14), the free energy derivatives with respect to the eight invariants depend on the network weights (Martonová et al. 2024), which we learn during neural network training.

### Neural network training

Using the explicit formulations for all Cauchy stress components, we train the network by optimizing a loss function *L* (see Eq. (15)), which penalizes the mean squared error in the $L_2$-norm between the modeled Cauchy stress $\varvec{\sigma }({\textbf{F}}_i,\varvec{w})$ and the measured Cauchy stress $\hat{\varvec{\sigma }}_i$, divided by the total number of data points $n_\mathrm{{data}}$. Equation (9) suggests that we can replace $w_\mathrm{{i,1}}$ and $w_\mathrm{{i,2}}$ by their product $w_\mathrm{{i,1}} w_\mathrm{{i,2}}$ for all odd-indexed weights $i=2n+1,n\ge 0$. This reduces the total number of non-negative trainable weights from 64 to 48, $\varvec{w}~=~\{w_\mathrm{{1,1}} w_\mathrm{{2,1}},w_\mathrm{{1,2}},w_\mathrm{{2,2}},\ldots ,w_\mathrm{{1,32}},w_\mathrm{{32,2}}\} \ge {\textbf{0}}$. The first-layer weights $w_\mathrm{{1,j}}$ are unit-less parameters, and the second-layer weights $w_\mathrm{{2,j}}$ have units of the stiffness. To promote model sparsity and enhance model interpretability, we add a $L_1$-regularization term $\alpha \, \Vert \, \varvec{w} \, \Vert _1$, to our loss function (McCulloch et al. 2023), leading to

15 $$\begin{aligned} L (\varvec{w} ; {\textbf{F}})&= \frac{1}{n_\mathrm{{data}}} \sum _{i=1}^{n_\mathrm{{data}}} \Vert \, \varvec{\sigma }({\textbf{F}}_i,\varvec{w}) - \hat{\varvec{\sigma }}_i \, \Vert _2^2 \nonumber \\&\quad + \alpha \, \sum _{i=1}^{n_{48}} \!\,|\, w_i \,| \rightarrow \text{ min }\,. \end{aligned}$$

In the following, we set the regularization parameter $\alpha = 0.01$, as proposed in recent studies (Martonová et al. 2024; Vervenne et al. 2025). To minimize the loss function in Eq. (15), we use the adaptive first-order gradient-based optimizer Adam (Kingma and Ba 2014). The network is trained for up to $30\,000$ epochs with a batch size of 32. We implement early stopping criterion if accuracy does not improve for $1\,000$ consecutive epochs. To mitigate the risk of convergence to local minima, we initialize the network weights randomly. Specifically, we use the Glorot normal initializer for layers with identity functions, and a random uniform initializer for layers with exponential functions, assigning non-negative weights with a maximum value of 0.1. We quantify model performance using the coefficient of determination $\mathrm R^2$, both individually for each individual experiment and combined across all experiments. Based on the finding in (Martonová et al. 2024) that training with only biaxial experimental data or with only triaxial shear data is not sufficient, we train our network on all available datasets. However, we note that excluding either a particular biaxial experiment or a particular triaxial shear experiment does not significantly influence the presented results.

## Results and discussion

To systematically investigate the influence of the input and output noise, we perform the following learning scenarios:

- we vary the amount of the Gaussian noise added the experimental data according to Eq. (8), and we assume perfect fiber, sheet and normal alignment ($\kappa _\textrm{f}=\kappa _\textrm{s}=\kappa _\textrm{n}=0$),
- we add 3% Gaussian noise to the experimental data and vary the dispersion in the fiber direction ($\kappa _\textrm{f}\ne 0$); we assume perfect alignment in sheet and normal directions ($\kappa _\textrm{s}=\kappa _\textrm{n}=0$),
- we add 3% Gaussian noise to the experimental data, and we vary the dispersion in the sheet and normal directions ($\kappa _\textrm{s}=\kappa _\textrm{n}\ne 0$); we assume perfect alignment in fiber direction ($\kappa _\textrm{f}=0$),
- we add 3% Gaussian noise to the experimental data, and we vary the dispersion in the fiber, sheet and normal directions ($\kappa _\textrm{f}=\kappa _\textrm{s}=\kappa _\textrm{n}\ge 0$),
- we add 3% Gaussian noise to the experimental data and treat $\kappa _\textrm{f}, \kappa _\textrm{s}, \kappa _\textrm{n}\ge 0$ as trainable parameters,
- we train the network without dispersion ($\kappa _\textrm{f}, \kappa _\textrm{s}, \kappa _\textrm{n}=0$) with simulated data obtained with varying dispersions in the fiber, sheet, and normal directions ($\kappa _\textrm{f}=\kappa _\textrm{s}=\kappa _\textrm{n}\ge 0$).

**Fig. 4:**
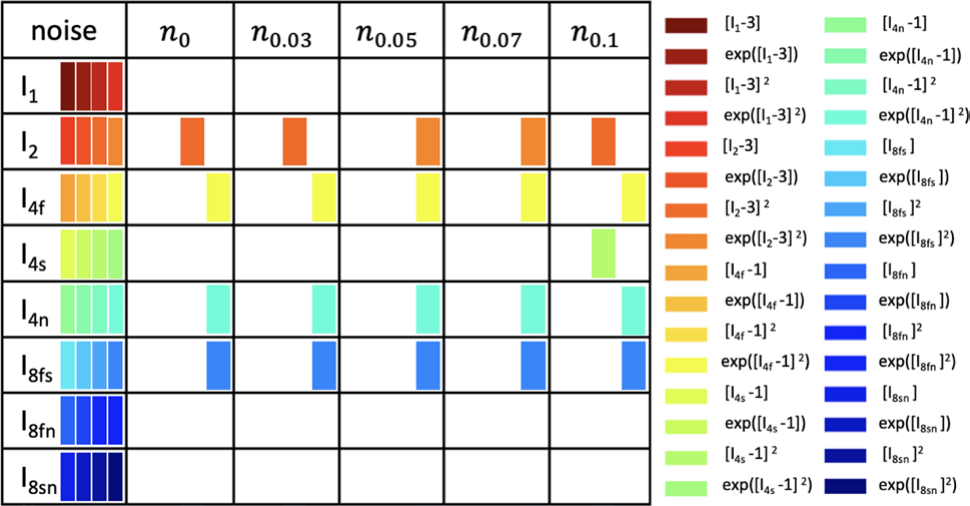
Model discovery for human myocardium with varying Gaussian noise. Active invariants for varying amount of Gaussian noise, 0%, 3%, 5%, 7%, 10%, added to the measured stress components according to Eq. (8); color-coded blocks represent the contributions of the particular active term out of four possible terms, depending on one of the eight possible invariants given in the first column

**Fig. 5:**
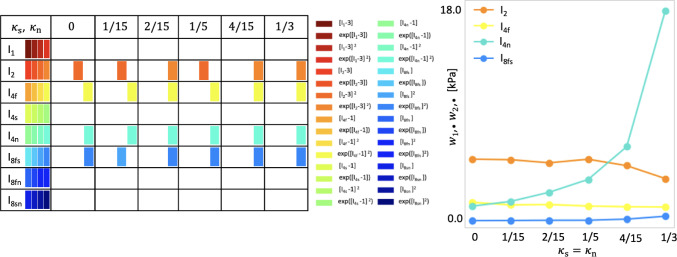
Model discovery for human myocardium with varying amount of dispersion in sheet and normal directions. Left: active invariants for varying amount of dispersion in sheet and normal directions, $\kappa _\textrm{s}=\kappa _\textrm{n}\in \{0,1/15,2/15,1/5,4/15,1/3\}, \kappa _\textrm{f}=0$; color-coded blocks represent the contributions of the particular active term out of four possible terms, depending on one of the eight possible invariants given in the first column. Right: network weight values in Eq. (9) that scale the contributions to the discovered free energy function $\psi$. The horizontal axis includes different values of dispersion; the vertical axis displays the sum of the products over the activated network weights according to $\sum _{i=4j}^{4j+4}w_{1,i}w_{2,i}$, where $j=1,2,4,5$ correspond to the terms depending on $I_2$ denoted by orange, $I_\mathrm{{4f}}$ denoted by yellow, $I_\mathrm{{4n}}$ denoted by green and $I_\mathrm{{8fs}}$ denoted by blue, respectively

**Fig. 6:**
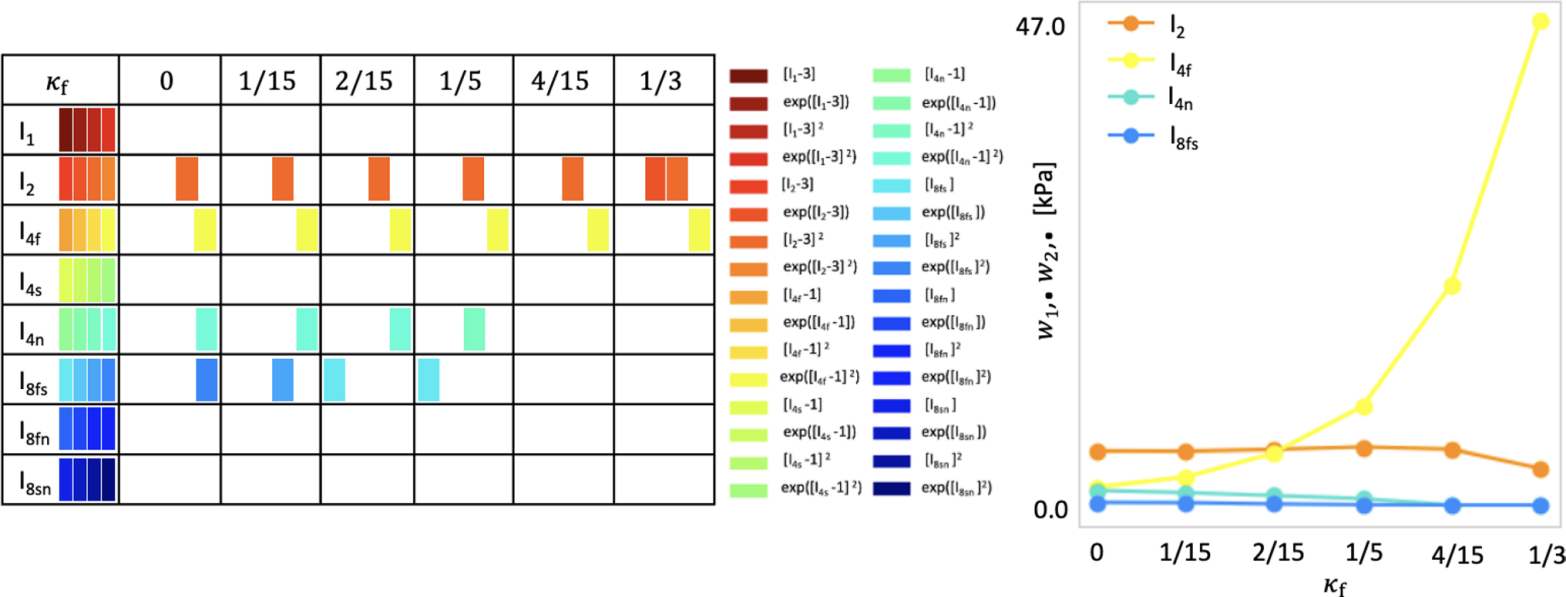
Model discovery for human myocardium with varying amount of dispersion in fiber direction. Left: active invariants for varying amount of dispersion in fiber directions, $\kappa _\textrm{f}\in \{0,1/15,2/15,1/5,4/15,1/3\}, \kappa _\textrm{s}=\kappa _\textrm{n}=0$; color-coded blocks represent the contributions of the particular active term out of four possible terms, depending on one of the eight possible invariants given in the first column. Right: network weight values in Eq. (9) that scale the contributions to the discovered free energy function $\psi$. The horizontal axis includes different values of dispersion; the vertical axis displays the sum of the products over the activated network weights according to $\sum _{i=4j}^{4j+4}w_{1,i}w_{2,i}$, where $j=1,2,4,5$ correspond to the terms depending on $I_2$ denoted by orange, $I_\mathrm{{4f}}$ denoted by yellow, $I_\mathrm{{4n}}$ denoted by green and $I_\mathrm{{8fs}}$ denoted by blue, respectively

**The network robustly discovers four-term models, even with aleatoric noise on the experimental data**. The robustness of our model discovery process is demonstrated by its insensitivity to the introduced random noise according to Eq. (8). As shown in Fig. 4, up to the data perturbations of 7% random Gaussian noise, our constitutive neural network consistently identifies similar four-term models and eight key parameters, with only minor variations. At the chosen regularization level of $\alpha =0.01$, the anisotropic invariants ${I}_\mathrm{{4f}}^*={I}_\mathrm{{4f}}, {I}_\mathrm{{4n}}^*={I}_\mathrm{{4n}}, I_\mathrm{{8fs}}$ contribute with an exponential quadratic term to the free energy $\psi$ and are highlighted in orange, yellow, turquoise, and blue in Fig. 4. For the isotropic second invariant $I_2$, the contributing terms are quadratic for noise levels less or equal to 3% and equal to 10%, whereas they are exponential quadratic for levels of noise equal to 5% and 7%. Notably, the data noise of 10% activates an additional invariant ${I}_\mathrm{{4s}}^*={I}_\mathrm{{4s}}$. Interestingly, this term is also present in the classical Holzapfel Ogden model for cardiac tissue (Holzapfel and Ogden 2009).

**Fig. 7:**
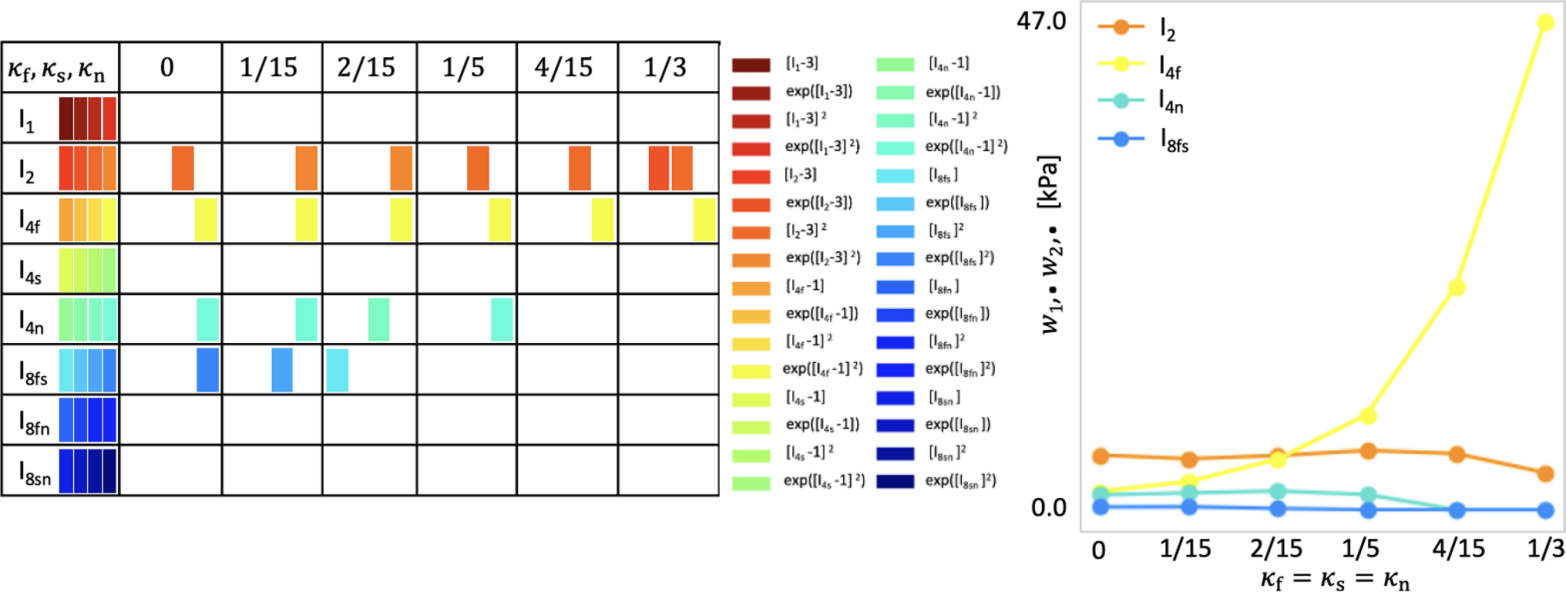
Model discovery for human myocardium with varying amount of dispersion in fiber, sheet, and normal directions. Left: active invariants for varying amount of dispersion in fiber, sheet, and normal directions, $\kappa _\textrm{f}=\kappa _\textrm{s}=\kappa _\textrm{n}\in \{0,1/15,2/15,1/5,4/15,1/3\}$; color-coded blocks represent the contributions of the particular active term out of four possible terms, depending on one of the eight possible invariants given in the first column. Right: network weight values in Eq. (9) that scale the contributions to the discovered free energy function $\psi$. The horizontal axis includes different values of dispersion; the vertical axis displays the sum of the products over the activated network weights according to $\sum _{i=4j}^{4j+4}w_{1,i}w_{2,i}$, where $j=1,2,4,5$ correspond to the terms depending on $I_2$ denoted by orange, $I_\mathrm{{4f}}$ denoted by yellow, $I_\mathrm{{4n}}$ denoted by green and $I_\mathrm{{8fs}}$ denoted by blue, respectively

**Fig. 8:**
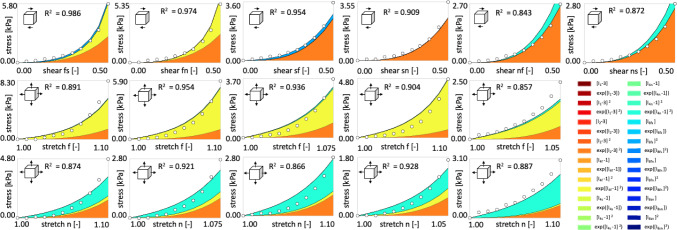
Human myocardial tissue data from triaxial shear and biaxial extension tests and the discovered model incorporating small dispersion in fiber, sheet, and normal directions. First row displays Cauchy stress components as functions of shear strains during triaxial shear tests; second and third rows display stretches during biaxial extension tests in fiber and normal directions, respectively. Experimental data from (Sommer et al. 2015), illustrated with dots, are used for training the network from Fig. 2 with dispersions in fiber, sheet, and normal direction $\kappa _\textrm{f}=\kappa _\textrm{s}=\kappa _\textrm{n}=1/15$. Color-coded areas indicate the activated terms, out of 32 possible terms displayed in the legend, that contribute to the stress components derived from the discovered free energy function $\psi$ according to Eq. (10)

**Fig. 9:**
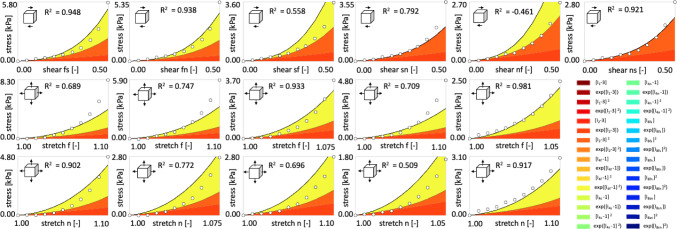
Human myocardial tissue data from triaxial shear and biaxial extension tests and the discovered model incorporating full dispersion in fiber, sheet, and normal directions. First row displays Cauchy stress components as functions of shear strains during triaxial shear tests; second and third rows display stretches during biaxial extension tests in fiber and normal directions, respectively. Experimental data from (Sommer et al. 2015), illustrated with dots, are used for training the network from Fig. 2 with dispersions in fiber, sheet, and normal direction $\kappa _\textrm{f}=\kappa _\textrm{s}=\kappa _\textrm{n}=1/3$. Color-coded areas indicate the activated terms, out of 32 possible terms displayed in the legend, that contribute to the stress components derived from the discovered free energy function $\psi$ according to Eq. (10)

**The network robustly discovers four-term models, even with dispersion in sheet and normal directions**. As highlighted in Fig. 5 left, for all variations in the dispersion parameters $\kappa _\textrm{s}$ and $\kappa _\textrm{n}$, we recover four-term models which depend on the four invariants $I_2, {I}_{4f}^*, {I}_{4n}^*$ and $I_{8fs}$. Except for the second invariant $I_2$ with the levels of dispersion $\kappa _\textrm{s}=\kappa _\textrm{n}\in \{0,1/15,1/5\}$ and the eight invariant $I_\mathrm{{8fs}}$ with $\kappa _\textrm{s}=\kappa _\textrm{n}=1/15$, both contributing quadratically in the free energy function (9), the active terms are exponential quadratic. The plot in Fig. 5 right indicates that, with increasing dispersion, the stiffness in the normal direction, quantified by the product of the network weights $w_{1,20}\,w_{2,20}$, increases. This is reasonable, since a dispersed normal direction will result in reduced contributions to the strain energy in the fn-plane during biaxial extension and in the fn- and sn-planes during shear testing. Interestingly, as suggested by the last row in Table 1, the mean goodness of fit slightly improves, from $\mathrm R^2=0.890$ for $\kappa _\textrm{f}=\kappa _\textrm{s}=\kappa _\textrm{n}=0$ to $\mathrm R^2=0.922$ for $\kappa _\textrm{s}=\kappa _\textrm{n}=4/15$. Strikingly, when $\kappa _\textrm{s}$ and $\kappa _\textrm{n}$ are close to 1/3, the dispersed invariants $I^*_\mathrm{{4s}}$ and $I^*_\mathrm{{4n}}$ given in (7) reduce to scalar multiples of $I_1$. As a result, the final strain energy function simplifies to one that depends almost entirely on $I_1$ and the primary fiber invariant $I_\mathrm{{4f}}$, eliminating any functional role for dispersion. This aligns closely with transversely isotropic models (Guccione, McCulloch, and Waldman 1991; Holzapfel and Ogden 2009; Gasser et al. 2006; Costa, Hunter, and McCulloch 1996).

**Model discovery is robust against small to moderate dispersions in fiber direction**. Figures 6 and 7 showcase that, for dispersion up to $\kappa _\textrm{f}=1/5$ with perfectly aligned sheet and normal directions and for the dispersion up to $\kappa _\textrm{f}=\kappa _\textrm{s}=\kappa _\textrm{n} =2/15$, we recover four-term models which depend on the four invariants $I_2, {I}_\mathrm{{4f}}^*, {I}_\mathrm{{4n}}^*$ and $I_\mathrm{{8fs}}$. The activated terms are predominantly quadratic and exponential quadratic, highlighting the high nonlinear material response. Notably, as shown in Tables 2 and 3 and Figs. 6 and 7 right, the weights scaling the linear terms depending on the eight invariant $I_\mathrm{{8fs}}$ are relatively small. With a higher regularization parameter $\alpha$, they would possibly be deactivated. High dispersions in fiber direction ($\kappa _\textrm{f}\ge 1/5$) induce model sparsity and reduces the goodness of fit. With increasing dispersion in fiber direction, we observe an increasing stiffness of the fiber, measured by the product $w_{1,12} \, w_{2,12}$, suggested by the yellow curve in Figs. 6 and 7 right. This nearly exponential increase can be explained by the softening effect of fiber dispersion (Melnik et al. 2018). In the extreme case of fully dispersed fibers ($\kappa _\textrm{f}=1/3$), the fiber contribution is spread out over the entire three-dimensional sphere as visualized in Fig. 3 right. Figures 8 and 9 depict the individual contributions of specific model terms to the modeled stress components. It is obvious that the assumed dispersion in fiber and sheet directions activates the invariant ${I}_\mathrm{{4n}}^*$ in the modeled fiber stress $\sigma _\mathrm{{ff}}$, see second row in the respective figures. Analogously, the invariant ${I}_\mathrm{{4f}}^*$ is activated when modeling the normal stress $\sigma _\mathrm{{nn}}$, see third row in the respective figures. For the full dispersion $\kappa _\textrm{f}=\kappa _\textrm{s}=\kappa _\textrm{n}$, both invariants, $I_\mathrm{{4f}}^*$ and $I_\mathrm{{4n}}^*$, contribute to the overall stress equally resulting in the equal modeled stresses $\sigma _\mathrm{{ff}}=\sigma _\mathrm{{nn}}$. We note that rescaling the stress axis in the second and third rows in Fig. 9 would result in the equal plots rows two and three. However, this equality does not correspond to the experimental data, which is as well reflected by the low $\mathrm R^2=0.722$ given in Table 3.

**Across all dispersion settings, the network consistently discovers models depending on the second invariant**. Interestingly, all discovered models for all levels of dispersion incorporate the second invariant rather than the first. As shown in Tables 1,  2 and 3, except for the full dispersion in the fiber direction, the discovered models rely exclusively on the quadratic term in the second invariant $I_2$. As visualized in Figs.  5, 6 and 7, the product of the discovered weights $w_{1,7} \, w_{2,7}$ for the solely quadratic term and $w_{1,8}w_{2,8}$ for the quadratic exponential term changes only minimally, with its minimum of 4.63 kPa for $\kappa _\textrm{s}=\kappa _\textrm{n}=4/15$ and maximum of 5.58 kPa $\kappa _\textrm{f}=\kappa _\textrm{s}=\kappa _\textrm{n}=1/5$. This selective preference is visually corroborated in Figs. 8 and 9, where the color-coded stress terms are dominated by orange contributions from the second invariant $I_2$. The dominance of the second invariant contrasts with the commonly used models that depend solely on the first invariant (Treloar 1948; Demiray 1976; Lanir 1983; Holzapfel and Ogden 2009; Budday et al. 2017; Guan et al. 2019), but it aligns well with a few previous models (Weiss et al. 1996; Horgan and Smayda 2012) and with recently discovered models (Kuhl and Goriely 2024; Linka and Kuhl 2024; Martonová et al. 2024; Vervenne et al. 2025) for soft biological tissues. Notably, while $I_1$ is quadratic in the principal stretches, $I_2$ is quartic, thereby providing a more sensitive measure of nonlinearity in finite strain regimes (Kuhl and Goriely 2024).

**Discovering of the most suitable dispersion parameter set**. Treating $\kappa _\textrm{f}$, $\kappa _\textrm{s}$, and $\kappa _\textrm{n}$ as non-negative trainable parameters, we obtain a lower bound for fiber dispersion, i.e., $\kappa _\textrm{f} = 0$, $\kappa _\textrm{s} = 0.154$, and $\kappa _\textrm{n} = 0.321$. This result aligns with our previous observation that improved goodness of fit is achieved for small to moderate dispersions in the fiber direction ($\kappa _\textrm{f} \le 2/15$) and arbitrary dispersions in the sheet and normal directions. For comparison, calibrating the Holzapfel Ogden model with dispersed invariants ${I}_\textrm{4f}^*$ and ${I}_\textrm{4s}^*$, a study reports dispersion parameters $\kappa _\textrm{f} = 0.00765$ and $\kappa _\textrm{s} = 0.0249$ (Eriksson et al. 2013), also suggesting that only small dispersions in the fiber direction are consistent with experimental data. However, using this set of dispersion parameters for our model discovery, we recover the same four-term model depending on the invariants $I_2$, ${I}_\textrm{4f}^*$, ${I}_\textrm{4n}^*$, and ${I}_\textrm{8fs}$. The corresponding goodness of fit, $\mathrm R^2 = 0.896$, increases slightly compared to the non-dispersed network ($\mathrm R^2 = 0.890$), but remains lower than for some particular settings of $\kappa _i$, $i \in \mathrm{f, \mathrm s, \mathrm n}$ (see Tables 1, 3, and 2). In particular, the most suitable dispersion parameter set mentioned above yields the highest $\mathrm R^2 = 0.925$. Taken together, these findings show that the discovered dispersion parameters reflect known aspects of myocardial microstructure. The model consistently identifies either perfect alignment or minimal dispersion in the fiber direction, consistent with the highly organized structure of cardiomyocytes. In contrast, moderate to high dispersion in the sheet and normal directions corresponds to the more variable organization of myocardial sheetlets. While assuming perfect fiber alignment can be a useful simplification, experimental studies consistently report small but nonzero fiber dispersion. For instance, diffusion tensor imaging and histological analyses reveal angular dispersions between $5^\circ$ and $15^\circ$, depending on transmural location and species (Scollan et al. 2000; Pashakhanloo et al. 2016). Thus, using the discovered four-term model with a small amount of dispersion in all three directions offers a favorable balance between physiological plausibility and model performance. These results suggest that the model implicitly captures structural anisotropy without requiring explicit microstructural input, potentially motivating targeted experiments such as diffusion tensor imaging or three-dimensional histological analysis to better quantify dispersion, particularly in the sheet and normal directions.

**Neural network without dispersion can accurately approximate a model with small dispersion**. Table 4 summarizes the discovered material parameters from training our constitutive neural network visualized in Fig. 2 without dispersion, ($\kappa _\textrm{f}=\kappa _\textrm{s}=\kappa _\textrm{n} = 0$) on simulated data that are generated with varying levels of fiber, sheet, and normal dispersions. In particular, for a small dispersion level $\kappa _\textrm{f}=\kappa _\textrm{s}=\kappa _\textrm{n} =1/15$, the network accurately approximates the data and rediscovers a four-term model from the previous study (Martonová et al. 2024) with high precision ($\mathrm R^2=0.998$). This indicates that a dispersion-free model may be sufficient in regimes with small dispersion, underscoring the robustness of automated model discovery. However, as the degree of dispersion increases, the network identifies different sets of active terms, suggesting that dispersion significantly influences the underlying mechanics. This highlights the importance of incorporating formulations with dispersion when modeling highly heterogeneous materials.

**Table 1:** Discovered mate﻿rial parameters for human myocardial tissue for varying amount of sheet and normal dispersions. Interpretable network weights for simultaneous training with six shear and five biaxial tests with a regularization parameter $\alpha =0.01$ for six different pairs of dispersion parameters $\kappa _\textrm{s}=\kappa _\textrm{n}\in \{0,1/15,2/15,1/5,4/15,1/3\}$, $\kappa _\textrm{f}=0$; mean goodness of fit $\text{ R}^2$

Network weights	Model term	${\varvec{\kappa _\textrm{s}=\kappa _\textrm{n}}}$					
$w_{1,\bullet }$ [–], $w_{2,\bullet }$ [kPa]	[–]	0	1/15	2/15	1/5	4/15	1/3
$w_{1,7} \cdot \, w_{2,7}\,$	$[I_{2}-3]^2$	5.153	5.108	–	5.144	–	–
$w_{1,8}, \,\, w_{2,8}\,$	$\exp ([I_{2}-3]^2)$	–	1.075, 4.520	–	–	1.215, 3.813	1.401, 2.572
$w_{1,12}, w_{2,12}$	$\exp ([{I}_\mathrm{{4f}}^*-1]^2)$	21.062, 0.081	23.844, 0.063	23.331,0.065	23.925, 0.059	24.687, 0.055	24.214, 0.055
$w_{1,20}, w_{2,20}$	$\exp ([I_\mathrm{{4n}}^*-1]^2)$	4.132, 0.340	4.683, 0.380	3.565, 0.705	2.598, 1.358	2.759, 2.228	4.044, 4.202
$w_{1,23} \cdot \, w_{2,23}$	$[I_\mathrm{{8fs}}]^2$	–	0.257	–	–	–	–
$w_{1,24}, w_{2,24}$	$\exp ([I_\mathrm{{8fs}}]^2)$	0.511, 0.485	–	0.517, 0.532	0.537, 0.514	0.605, 0.607	1.696, 0.351
$\text{ R}^2$		0.890	0.898	0.905	0.914	0.922	0.920

**Table 2:** Discovered material parameters for human myocardial tissue for varying amount of fiber dispersion. Interpretable network weights for simultaneous training with six shear and five biaxial tests with a regularization parameter $\alpha =0.01$ for six different dispersion parameters $\kappa _\textrm{f}\in \{0,1/15,2/15,1/5,4/15,1/3\}$, $\kappa _\textrm{s}=\kappa _\textrm{n}=0$; mean goodness of fit $\text{ R}^2$

Network weights	Model term	${\varvec{\kappa _\textrm{f}}}$					
$w_{1,\bullet }$ [–], $w_{2,\bullet }$ [kPa]	[–]	0	1/15	2/15	1/5	4/15	1/3
$w_{1,6}, \,\, w_{2,6}\,$	$\exp [I_{2}-3]$	–	–	–	–	–	0.629, 0.721
$w_{1,7} \cdot \, w_{2,7}\,$	$[I_{2}-3]^2$	5.153	5.121	5.290	5.527	5.308	3.080
$w_{1,12}, w_{2,12}$	$\exp ([{I}_\mathrm{{4f}}^*-1]^2)$	21.062, 0.081	25.558, 0.105	24.893, 0.199	23.176, 0.406	14.619, 1.438	5.059, 9.141
$w_{1,19} \cdot \, w_{2,19}$	$[{I}_\mathrm{{4n}}^*-1]^2$	–	–	–	0.608	–	–
$w_{1,20}, w_{2,20}$	$\exp ([{I}_\mathrm{{4n}}^*-1]^2)$	4.132, 0.340	5.517, 0.214	5.644, 0.161	–	–	–
$w_{1,21} \cdot \, w_{2,21}$	$[I_\mathrm{{8fs}}]$	–	–	0.104	0.035	–	–
$w_{1,23} \cdot \, w_{2,23}$	$[I_\mathrm{{8fs}}]^2$	–	0.222	–	–	–	–
$w_{1,24}, w_{2,24}$	$\exp ([I_\mathrm{{8fs}}]^2)$	0.511, 0.485	–	–	–	–	–
$\text {R}^2$		0.890	0.905	0.904	0.885	0.852	0.720

**Table 3:** Discovered material parameters for human myocardial tissue for varying amount of fiber, sheet and normal dispersions. Interpretable network weights for simultaneous training with six shear and five biaxial tests with a regularization parameter $\alpha =0.01$ for six different triples of dispersion parameters $\kappa _\textrm{f}=\kappa _\textrm{s}=\kappa _\textrm{n}\in$ $\{0,1/15,2/15,1/5,4/15,1/3\}$; mean goodness of fit $\text{ R}^2$

network weights	model term	${\varvec{\kappa _\textrm{f}=\kappa _\textrm{s}=\kappa _\textrm{n}}}$					
$w_{1,\bullet }$ [–], $w_{2,\bullet }$ [kPa]	[–]	0	1/15	2/15	1/5	4/15	1/3
$w_{1,6}, \,\, w_{2,6}\,$	exp$[I_{2}-3]$	–	–	–	–	–	0.642, 0.740
$w_{1,7} \cdot \, w_{2,7}\,$	$[I_{2}-3]^2$	5.153	–	–	5.579	5.303	3.026
$w_{1,8}, \,\, w_{2,8}\,$	$\exp ([I_{2}-3]^2)$	–	1.112,4 4.333	1.222, 4.179	–	–	–
$w_{1,12}, w_{2,12}$	$\exp ([{I}_\mathrm{{4f}}^*-1]^2)$	21.062, 0.081	25.315, 0.105	25.442, 0.187	24.149, 0.370	14.439, 1.459	5.359, 8.582
$w_{1,19} \cdot \, w_{2,19}$	$[{I}_\mathrm{{4n}}^*-1]^2$	–	–	1.755	–	–	–
$w_{1,20}, w_{2,20}$	$\exp ([{I}_\mathrm{{4n}}^*-1]^2)$	4.132, 0.340	5.532, 0.286	–	1.393, 1.030	–	–
$w_{1,23} \cdot \, w_{2,23}$	$[I_\mathrm{{8fs}}]^2$	–	0.270	–	–	–	–
$w_{1,24}, w_{2,24}$	$\exp ([I_\mathrm{{8fs}}]^2)$	0.511, 0.485	–	–	–	–	–
$\text{ R}^2$		0.890	0.910	0.904	0.888	0.854	0.722

**Table 4:** Discovered material parameters for human myocardial tissue trained on simulated data with fiber, sheet and normal dispersions. Interpretable network weights for simultaneous training with simulated data using models with fiber, sheet and normal dispersion for six shear and five biaxial experiments. For the training, neural network from Fig. 2 with $\kappa _\textrm{f}=\kappa _\textrm{s}=\kappa _\textrm{n}=0$ and regularization parameter $\alpha =0.01$ is utilized; mean goodness of fit $\text{ R}^2$

Network weights	Model term	Dispersion in simulated training data $\kappa _\textrm{f} = \kappa _\textrm{s} = \kappa _\textrm{n}$					
$w_{1,\bullet }$ [–], $w_{2,\bullet }$ [kPa]	[–]	0	1/15	2/15	1/5	4/15	1/3
$w_{1,5} \cdot w_{2,5}$	$[I_{2}-3]$	–	–	–	–	–	0.570
$w_{1,6}, \,\, w_{2,6}\,$	$\exp [I_{2}-3]$	–	–	–	0.517, 0.504	–	
$w_{1,7} \cdot \, w_{2,7}$	$[I_{2}-3]^2$	–	–	–	0.066	5.700	2.688
$w_{1,8} \cdot \, w_{2,8}$	$\exp ([I_{2}-3]^2)$	1.864, 2.575	2.440, 1.901	1.847, 2.749	–	–	–
$w_{1,10}, w_{2,10}$	$\exp ([{I}_\mathrm{{4f}}-1])$	–	–	–	–	–	0.249, 0.246
$w_{1,12}, w_{2,12}$	$\exp ([{I}_\mathrm{{4f}}-1]^2)$	18.758, 0.101	23.695, 0.120	11.105, 0.214	2.578, 1.086	6.144, 0.899	4.089, 11.188
$w_{1,18}, w_{2,18}$	$\exp ([{I}_\mathrm{{4n}}-1])$	–	–	0.136, 0.136	–	0.303, 0.303	–
$w_{1,19} \cdot \, w_{2,19}$	$[{I}_\mathrm{{4n}}-1]^2$	–	–	1.480	1.064	0.040	–
$w_{1,20}, w_{2,20}$	$\exp ([{I}_\mathrm{{4n}}-1]^2)$	1.494, 1.026	1.996, 0.885	–	–	–	–
$w_{1,24}, w_{2,24}$	$\exp ([I_\mathrm{{8fs}}]^2)$	0.497, 0.485	0.496, 0.472	0.517, 0.460	–	0.980, 0.341	–
$\text{ R}^2$		0.999	0.998	0.981	0.918	0.899	0.997

**Limitations and outlook**. First, our current model assumes full incompressibility, which may not fully represent the mechanical behavior of myocardial tissue that exhibits slight compressibility (Bonnemains et al. 2018; Avazmohammadi et al. 2020). Future research could explore a nearly incompressible formulation by incorporating an additional trainable parameter representing the bulk modulus. Second, fiber dispersion is represented exclusively through the fourth invariant, which may limit the model’s ability to capture more complex anisotropic effects. Although we lack of experimental data regarding the dispersion in the coupling invariants, future modification of the coupling invariants is possible (Melnik et al. 2018). Third, our training was conducted on a limited dataset. Efforts to obtain or simulate broader datasets for human myocardium would improve the robustness of model discovery. While the dataset could potentially be enriched with more recent experimental data obtained from animal models (Li et al. 2021; Ahmad et al. 2022; Kakaletsis et al. 2023), further investigation is needed to assess the extent to which such data can be reliably translated to human cardiac models (Martonová et al. 2021). Lastly, the model focuses exclusively on passive mechanical behavior, without explicit distinction between active and passive responses or consideration of biochemical feedback mechanisms. Future work can address the inclusion of active mechanical responses, crosslink dynamics, and reaction-diffusion processes (Göktepe and Kuhl 2010; Gizzi et al. 2017, Pandolfi et al. 2016, 2017a, b).

## Conclusion

In this work, we investigated the effects of fiber, sheet, and normal dispersions and random noise on the measured data in passive material model discovery using an orthotropic perfectly incompressible constitutive neural network for human myocardium. Overall, despite the non-convex nature of the minimization problem, our approach consistently identifies similar material models. This result underscores the robustness and reliability of the automated model discovery. Added random noise up to 3% has no influence on the discovered model. Up to a moderate dispersion in the fiber direction ($0<\kappa _\textrm{f} \le 2/15$), an arbitrary dispersion in the sheet and normal directions ($\kappa _\textrm{s}=\kappa _\textrm{s} > 0$) with perfectly aligned fibers ($\kappa _\textrm{f} =0$), or a small equal dispersion ($\kappa _\textrm{f}=\kappa _\textrm{s}=\kappa _\textrm{n} =1/15$), all improve the goodness of fit and recover our previously discovered four-term model, subject to the isotropic invariant $I_2$, the dispersed fourth invariants $I_\mathrm{{4f}}^*$, $I_\mathrm{{4n}}^*$, and the coupling invariant $I_\mathrm{{8fs}}$ in their quadratic or quadratic exponential forms. Introducing a constitutive neural network that accounts for dispersion in three directions enables us to account for the micro-structural architecture, to discover the best dispersion parameters, and to interpret model sensitivity with respect to the fiber, sheet, and normal directions.

## Supplementary information

Our source code, data, and examples are available at https://github.com/LivingMatterLab/CANN.

## Data Availability

No datasets were generated or analyzed during the current study.
